# Resource management and clinical outcomes during a national intravenous fluid shortage

**DOI:** 10.1002/jhm.70352

**Published:** 2026-05-15

**Authors:** Stacy A. Johnson, Erin R. Fox, Smitha Warrier, Russell Vinik

**Affiliations:** ^1^ Department of Internal Medicine, Division of General Internal Medicine University of Utah School of Medicine Salt Lake City Utah USA; ^2^ Department of Pharmacy University of Utah Health Salt Lake City Utah USA; ^3^ Department of Anesthesiology University of Utah School of Medicine Salt Lake City Utah USA

## Abstract

**Background:**

A natural disaster, Hurricane Helene, damaged a production facility supplying 60% of intravenous (IV) fluids in the United States. A nationwide IV fluid shortage followed.

**Objectives:**

To evaluate a multifaceted IV fluid conservation intervention's effect on IV fluid utilization and clinical outcomes during the shortage.

**Methods:**

Retrospective, quasi‐experimental study using interrupted time series and multivariable regerssion at the University of Utah Hospital, an 817‐bed academic medical center. Inpatient admissions to surgical, medical, or obstetrics and gynecology services between July 1, 2024 and January 1, 2025 were included. The intervention comprised system‐wide communications, health record modifications, leadership meetings, and real‐time monitoring. The primary outcome was IV fluid utilization; secondary outcomes were length of stay, diuretic administration, acute kidney injury, renal replacement therapy, ICU transfer, hypotension, and inpatient and 30‐day mortality.

**Results:**

A total of 15,847 admissions were analyzed, including 8164 preintervention and 7683 postintervention admissions. Mean age was 52.9 years; 8621 (54.4%) were female. IV fluid utilization decreased from a mean (95% confidence interval [CI]) of 5.5 (5.3–5.7) bags to 3.5 (3.4–3.6) bags per admission following the intervention (*p* < .001). Length of stay decreased by 0.6 days postintervention (*p* < .001). Loop diuretic administration decreased (odds ratio [OR] = 0.87 [0.79−0.95]; *p* = .001), and an increase in acute kidney injury was observed (OR = 1.1 [1.0−1.3]; *p* = .024). No significant differences were observed for renal replacement therapy, ICU transfer, hypotension, or mortality outcomes.

**Conclusions:**

Reductions in IV fluid utilization, diuretic administration, and length of stay were observed following an IV fluid conservation intervention. Our strategies offer a framework for health systems facing similar shortages with the potential to improve clinical outcomes.

## INTRODUCTION

On September 24, 2024, Hurricane Helene damaged Baxter International Inc.'s facility in North Carolina, which supplied approximately 60% of intravenous (IV) fluids in the United States.^1^ Baxter subsequently placed all fluids on allocation, halted some shipments, and recommended clinicians initiate measures to conserve IV fluid use.^2^

University of Utah Healthcare has tracked national drug shortages since 2001, and this shortage represented the tenth severe shortage of IV fluids, such as 0.9% sodium chloride (saline), to affect patient care. Previous fluid shortages generally involved a single product type.^3^ In contrast, the shortage described here involved concurrent shortages of both small‐ and large‐volume products as well as irrigation solutions, which further complicated conservation efforts. Despite the persistent nature of drug shortages, limited data have described associated patient outcomes.^4^

In response to the fluid shortage, our healthcare system implemented conservation efforts consistent with national recommendations.^5^ In this study, our primary aim was to compare IV fluid utilization before and after implementation of local conservation efforts, and our secondary aim was to evaluate associated clinical outcomes.

## METHODS

### Study design

We performed a quasi‐experimental, pre‐/postintervention study to compare resource utilization and clinical outcomes of hospitalized patients before and after implementation of fluid conservation efforts. The study was reviewed and deemed exempt by the University of Utah Institutional Review Board (IRB_00187062).

### Setting and participants

The study was conducted at the University of Utah Hospital, an 817‐bed academic medical center. All patients admitted as inpatient status were eligible for inclusion. Patients were assigned to one of two cohorts, defined as preintervention and postintervention, depending on whether the admission began before or after the intervention date. The hospital service line was assigned based on the department and division of the assigned attending physician at the time of discharge. Prespecified subgroups (medical, surgical, and obstetric/gynecological [ob/gyn]) were defined based on the expected majority of hospitalizations with IV fluid administration. Patients were excluded if they received care exclusively in an emergency department without inpatient admission (*n* = 9), were discharged from an inpatient psychiatry (*n* = 1991) or physical medicine and rehabilitation service (*n* = 425), were born during the hospitalization (*n* = 2692), or the discharging service line was missing (*n* = 742). Subgroup assignment by service line is summarized in Supporting Information S1: Appendix A.

An electronic database query was designed to identify all inpatients admitted between July 1, 2024, and January 1, 2025. This study period allowed the creation of a 3‐month preintervention and a 3‐month postintervention period for comparisons.

### Study intervention

A multifaceted fluid conservation intervention was implemented on October 1, 2024. The intervention strategy was conceived by hospital leadership and broadly consisted of (1) systemwide communications describing the fluid shortage; (2) modification of the electronic health record (EHR) to support recommended conservation measures; and (3) strategic meetings with service line directors to further identify and disseminate conservation plans. An online dashboard was created with input from information technology, pharmacy, hospital operations, and supply chain leadership to allow real‐time monitoring of fluid product supply and usage (Supporting Information S1: Appendix B).

Communication about the shortage and interventions was essential to ensure systemwide action. A dedicated intranet site accessible to all staff was established on October 10, 2024. This site contained status updates and listed recommended conservation measures. An organization‐wide livestream also highlighted the shortage and conservation measures on October 16, 2024. Email communications to every clinician occurred eight times during the study period. A dedicated interdisciplinary team, including operations and clinical leaders, met frequently during the shortage, a total of 33 times. The real‐time supply dashboard tracked both usage and supply on hand to help facilitate decision‐making regarding conservation efforts.

The Pharmacy and Therapeutics Committee approved conservation measures on October 7, 2024, recommended in national shortage management guidelines co‐authored by University of Utah.^5^ These included recommendations to use oral hydration as an informal alternative to IV fluids when clinically appropriate, avoid using fluids to “keep vein open,” evaluate the clinical need for IV fluids at every shift change, and to avoid waste (Supporting Information S1: Appendix C). All EHR order sets were reviewed by October 8, 2024, to remove preselected orders for IV fluid administration (e.g., maintenance IV fluid in the perioperative period). On October 8, 2024, an alert was also developed in the EHR to alert providers ordering fluids for patients with oral diet orders or rates < 50 mL/h. This was later adjusted on November 27, 2024, to exclude ICU patients and selected order sets (e.g., diabetic ketoacidosis).

Service line directors provided additional conservation measures. For example, beginning October 8, 2024, patients undergoing low‐risk cataract surgery had an IV line with heparin lock rather than IV fluids running. On October 18, 2024, the infection prevention and control team recommended extending IV fluid hang times to 96 h for fluids without additives. The labor and delivery team decreased their standard IV fluid rates from 125 to 50 mL/h. The pharmacy department made multiple changes during October and November 2024, such as using premixed commercial products or changing standard doses to reduce the use of small‐volume bags.

### Study outcomes

Study outcomes were selected to first assess the effectiveness of the intervention on resource utilization, and then to compare clinical outcomes potentially impacted by the fluid shortage. The primary study outcome was the mean number of IV fluid bags dispensed during an admission. We chose to use the count of bags dispensed rather than the volume of IV fluid received, as once a bag of IV fluid is dispensed, it cannot be reallocated, regardless of the volume of fluid utilized. Secondary outcomes were defined as hospital length of stay, loop diuretic administration during an admission, count of days a loop diuretic was administered, cumulative dose of loop diuretic received, average daily dose of loop diuretic received, development of acute kidney injury (defined as a rise in serum creatinine ≥ 0.3 mg/dL within 48 h during admission),^6^ severe acute kidney injury (defined as a serum creatinine ≥ 4.0 mg/dL during admission),^6^ renal replacement therapy, transfer from an acute care unit to an intensive care unit (ICU), inhospital mortality, and 30‐day mortality.

Loop diuretic use was chosen as a surrogate marker of volume overload as clinical signs of volume overload are not reliably captured in the EHR. Furthermore, as loop diuretics are the mainstay of treatment for volume overload, other diuretic classes (i.e., thiazides and mineralocorticoid receptor antagonists) were not analyzed. To account for differences in potency between loop diuretics, all dosages were converted to oral furosemide equivalent units (Supporting Information S1: Appendix D).

Lastly, postoperative atrial fibrillation was assessed within the surgical subgroup, given the relationship between volume status and postoperative atrial fibrillation. Although postoperative atrial fibrillation has multiple triggers, significant differences in volume status between periods could produce a measurable difference.

### Statistical methods

An interrupted time series (ITS) analysis was used to evaluate the effectiveness of the intervention on IV fluid utilization. This quasi‐experimental design estimates both immediate change and changes in trends over time. An ITS model was constructed for the count of IV fluid bags dispensed, summarized as weekly averages. The Cumby–Huizinga test for autocorrelation was applied with six lags.^7^

To assess for differences in baseline characteristics between study cohorts, Student's *t*‐tests were applied to continuous variables and Pearson's *χ* ^2^ tests for categorical variables. Unadjusted analysis of study outcomes was performed with Student's *t‐*test and Pearson's *χ* ^2^ test, where appropriate. To adjust for potential confounding, generalized linear models (GLMs) with a gamma distribution and log‐link function were constructed for continuous outcomes, including bags of IV fluid, hospital length of stay, days of loop diuretic administration, and total and average daily dose of loop diuretic administration. Covariate selection was based on previously reported associations with study outcomes.^8^, ^9^, ^10^, ^11^, ^12^ Covariates included in the models were age, gender, body mass index (BMI), Charlson comorbidity index, Medicare Severity Diagnosis Related Group (MS‐DRG) weight, and an indicator variable for the intervention period. Adjusted continuous outcomes were estimated using marginal effects at the means.^13^ Binary outcomes, including loop diuretic use, acute kidney injury, renal replacement therapy, hypotension, ICU transfer, postoperative atrial fibrillation, inhospital mortality, and 30‐day mortality, were assessed with multivariable logistic regression models. Odds ratios with corresponding 95% confidence intervals were reported. A sensitivity analysis was performed by comparing clinical outcomes from 1 year prior (July 1, 2023, to January 1, 2024) to the study period (July 1, 2024, to January 1, 2025) to evaluate for trends in outcomes attributable to seasonal^14^, ^15^ or other unmeasured confounders (e.g., start of academic year with new graduate medical education trainees—“July effect”).^16^ Lastly, a prespecified subgroup analysis was performed to compare outcomes within surgical, medical, and ob/gyn admissions. All statistical tests were two‐tailed, and a *p*‐value below 0.05 was considered significant. Analyses were performed using Stata/IC version 16.1 (StataCorp).

## RESULTS

### Study population

A total of 22,358 hospital admissions were identified during the study period. After applying exclusion criteria, 15,847 admissions remained (Supporting Information S1: Appendix E). The preintervention cohort included 8164 admissions, and the postintervention cohort included 7683 admissions. Baseline characteristics are reported in Table 1. A small difference in mean age (52.6 years vs. 53.3 years; *p* = .03) was present, with the postintervention cohort approximately 0.7 years older. Female gender was similar between preintervention (54.8%) and postintervention (54.0%) cohorts (*p* = .297). The majority of patients were White (~74%), and approximately 10% identified as Latin‐x in both cohorts. Medical admissions accounted for more than half of all admissions (52%–53%), and surgical admissions accounted for nearly one‐third of admissions (31%–33%). BMI, Charlson comorbidity index, and MS‐DRG weight (case mix index) were not significantly different between cohorts.

**Table 1:** Baseline patient characteristics.

Characteristic	Preintervention period	Postintervention period	*p* value
	(*N* = 8164)	(*N* = 7683)	
Age in years, mean (SD)	52.6 (19.7)	53.3 (19.4)	.03
Female, *n* (%)	4474 (54.8)	4147 (54.0)	.297
Race/ethnicity, *n* (%)			.001
White/Caucasian	6049 (74.4)	5702 (74.5)	
Latin‐x/Hispanic	887 (10.9)	813 (10.6)	
Black/African American	212 (2.6)	175 (2.3)	
Unidentified	325 (4.0)	395 (5.2)	
BMI in kg/m^2^, mean (SD)	29.0 (7.9)	29.2 (7.7)	.146
Admission type, *n* (%)			.028
Medical	4325 (53.0)	3996 (52.0)	
Surgical	2550 (31.2)	2544 (33.1)	
Ob/Gyn	1289 (15.8)	1143 (14.9)	
Charlson Comorbidity Index, mean (SD)	3.2 (3.3)	3.2 (3.2)	.767
MS‐DRG weight, mean (SD)	2.2 (2.9)	2.2 (2.8)	.499

Abbreviations: BMI, body mass index; MS‐DRG, Medicare Severity Diagnosis Related Group; Ob/Gyn, Obstetrics and Gynecology; SD, standard deviation.

### Unadjusted outcomes

A significant reduction in the number of dispensed IV fluid bags was observed following the intervention (mean [95% CI]; 6.0 [5.8–6.3] bags vs. 3.8 [3.6–3.9] bags; *p* < .001). Results of the ITS analysis are displayed in Figure 1, demonstrating a significant postintervention reduction over time (*ß*_3_ = −0.11 [−0.19, −0.2]; *p* = .014). The mean (95% CI) length of stay was significantly reduced after the intervention (5.8 [5.6–5.9] days vs. 5.1 [5.1–5.2] days; *p* < .001). Loop diuretic administration occurred less often during postintervention admissions (19.1% vs. 17.4%; *p* = .007). The duration of loop diuretic administration was significantly shorter in postintervention admissions (mean [95% CI], 0.91 [0.84–0.98] days vs. 0.78 [0.72–0.83] days; *p* = .025). The total dose of diuretic received, reported as oral furosemide equivalent units (FEU), was similarly reduced among postintervention admissions (mean [95% CI] FEU; 115.8 [101.3–130.3] FEU vs. 97.3 [84.3–104.6] FEU; *p* = .019). For the outcomes of acute kidney injury, renal replacement therapy, hypotension, ICU transfer, and inhospital and 30‐day mortality, no significant differences were observed between cohorts (Table 2). Among surgical admissions, the incidence of postoperative atrial fibrillation was not significantly different between pre‐ and postintervention periods (4.9% vs. 4.2%; *p* = .207).

**Figure 1:**
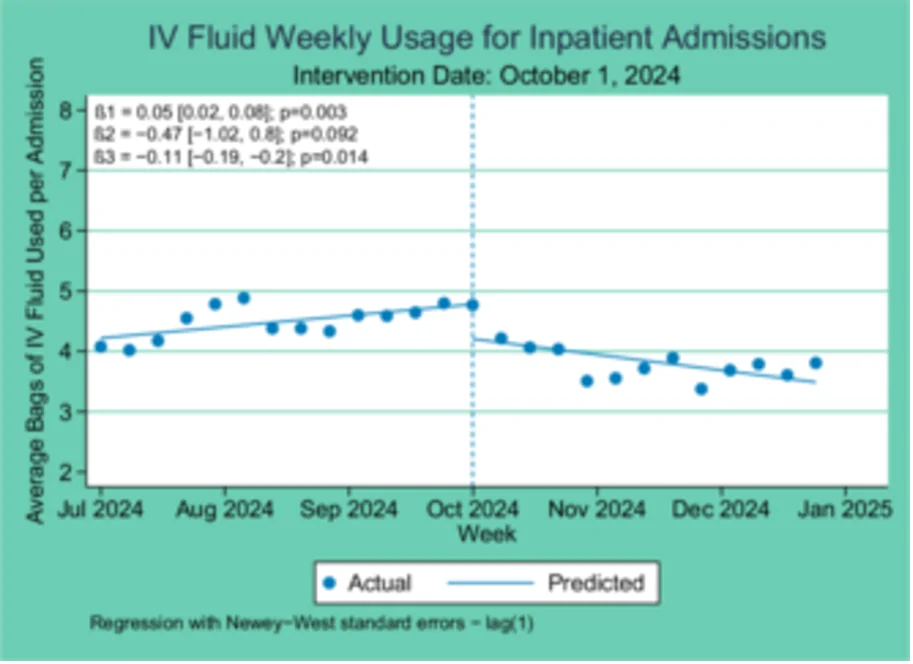
Interrupted time series analysis of weekly IV fluid usage. ß_1 _= preintervention weekly trend; ß_2_ = level change at time of intervention; ß_3_ = postintervention weekly trend. IV, intravenous

**Table 2:** Unadjusted clinical outcomes.

Outcome	Preintervention period	Postintervention period	*p* value
	(*n* = 8164)	(*n* = 7683)	
Resource utilization			
Bags of IV fluid dispensed, mean (95% CI)	6.0 (5.8–6.3)	3.8 (3.6–3.9)	<.001
Clinical outcomes			
Length of stay in days, mean (95% CI)	5.8 (5.6–5.9)	5.1 (5.0–5.2)	<.001
Loop diuretic administered, *n* (%)	1558 (19.1)	1339 (17.4)	.007
Days of diuretic administration, mean (95% CI)	0.91 (0.84–0.98)	0.78 (0.72–0.83)	.025
Total dose of diuretic administered in oral furosemide equivalents, mean (95% CI)	115.8 (101.3–130.3)	97.3 (84.3–104.6)	.019
Average daily dose of diuretic, mean (95% CI)	18.2 (17.1–19.4)	16.7 (15.5–17.8)	.056
Acute kidney injury, *n* (%)	790 (11.0)	816 (12.0)	.084
Severe acute kidney injury, *n* (%)	316 (4.4)	265 (3.9)	.118
Renal replacement therapy, *n* (%)	101 (1.2)	79 (1.0)	.215
Hypotension, *n* (%)	2496 (30.6)	2327 (30.3)	.696
ICU transfer, *n* (%)	312 (3.8)	269 (3.5)	.283
Postoperative atrial fibrillation^a^, *n* (%)	125 (4.9)^a^	106 (4.2)^a^	.207
30‐day mortality, *n* (%)	364 (4.5)	326 (4.2)	.507
Inhospital mortality, *n* (%)	195 (2.4)	176 (2.3)	.684

Abbreviations: CI, confidence interval; ICU, intensive care unit; IV, intravenous.

^a^ Analysis restricted to surgical admissions only. Preintervention denominator, *n* = 2550; postintervention denominator, *n* = 2544.

### Adjusted outcomes

Following multivariable adjustment for the prespecified covariates, the mean number of IV fluid bags dispensed decreased on average by two bags per admission during the postintervention period (mean [95% CI], preintervention 5.5 [5.3–5.7] bags vs. postintervention 3.5 [3.4–3.6] bags; *p* < .001). The mean length of stay decreased by 0.6 days following the intervention (mean [95% CI], 5.5 [5.4–5.6] days vs. 4.9 [4.8–5.0]; *p* < .001). Loop diuretic administration decreased during the postintervention period (odds ratio [OR] = 0.87 [0.79–0.95]; *p* = .001), although significant reductions in the total dose and duration were not observed. The odds of developing acute kidney injury were slightly increased (OR = 1.1 [1.0–1.3]; *p* = .024) following the intervention, although the need for renal replacement therapy was not significantly different (OR = 0.91; *p* = .625). For the outcomes of hypotension, ICU transfer, postoperative atrial fibrillation, and inhospital and 30‐day mortality, no significant differences were identified in the logistic regression models (Table 3).

**Table 3:** Adjusted clinical outcomes.

Outcomes	Preintervention period	Postintervention period	*p* value
	(*n* = 8164)	(*n* = 7683)	
Resource utilization			
Bags of IV fluid dispensed, mean (95% CI)	5.5 (5.3–5.7)	3.5 (3.4–3.6)	<.001
Clinical outcomes			
Length of stay in days, mean (95% CI)	5.5 (5.4–5.6)	4.9 (4.8–5.0)	<.001
Loop diuretic administered, OR (95% CI)	ref	0.87 (0.79–0.95)	.001
Days of diuretic administration, mean (95% CI)	0.57 (0.52–0.62)	0.50 (0.45–0.55)	.053
Total dose of diuretic administered in oral furosemide equivalents, mean (95% CI)	60.9 (53.0–68.7)	53.5 (45.2–61.9)	.207
Average daily dose of diuretic, mean (95% CI)	12.2 (11.2–13.3)	10.8 (9.8–11.8)	.057
Acute kidney injury, OR (95% CI)	ref	1.1 (1.0–1.3)	.024
Severe acute kidney injury, OR (95% CI)	ref	0.91 (0.77–1.08)	.299
Renal Replacement Therapy, OR (95% CI)	ref	0.92 (0.66–1.28)	.625
Hypotension, OR (95% CI)	ref	1.0 (0.9–1.1)	.983
ICU transfer, OR (95% CI)	ref	0.91 (0.67–1.24)	.564
Post‐operative atrial fibrillation^a^, OR (95% CI)	ref	0.87 (0.65–1.16)	.347
30‐day mortality, OR (95% CI)	ref	0.94 (0.80–1.11)	.495
Inhospital mortality, OR (95% CI)	ref	0.97 (0.78–1.20)	.767

Abbreviations: CI, confidence interval; ICU, intensive care unit; IV, intravenous; OR, odds ratio.

^a^ Analysis restricted to surgical admissions only. Preintervention denominator, *n* = 2550; postintervention denominator, *n* = 2544.

### Subgroups

Surgical admissions were associated with the greatest reductions in IV fluid utilization during the postintervention period, with a mean difference of −2.7 bags per admission (95% CI: −3.1 to −2.4; *p* < .001). The intervention appeared to be effective across different service lines with mean differences of −1.7 bags for medical admissions (95% CI: −2.0 to −1.4; *p* < .001) and −1.5 bags for ob/gyn admissions (95% CI: −1.7 to −1.3; *p* < .001). Surgical admissions were approximately 0.6 days shorter (*p* < .001) and medical admissions were 0.5 days shorter (*p* < .001) following the intervention. Among ob/gyn admissions, no significant changes in mean length of stay were observed after the intervention (3.1 days vs. 3.0 days; *p* = .735). Significant reductions in any diuretic use, total dose, average daily dose, and total duration were observed within the surgical subgroup following the intervention (Supporting Information S1: Appendix F). Among medical admissions, diuretic utilization, dose, and duration did not significantly change following the intervention. A very small reduction in diuretic duration was observed among ob/gyn admissions (0.03 days vs. 0.002 days; *p* = .032). No significant postintervention differences in acute kidney injury, hypotension, ICU transfer, or mortality were observed within the subgroups.

## DISCUSSION

In this quasi‐experimental study, we analyzed the effects of a multifaceted intervention for the conservation of IV fluids following a nationwide drug shortage. We observed significant postintervention reductions in IV fluid utilization and loop diuretic administration across multiple service lines, with an unanticipated reduction in length of stay. Although a small increased risk of acute kidney injury was identified, other clinical outcomes remained stable. Importantly, these findings strengthen the available evidence on clinical outcomes during a drug shortage and warrant further discussion.

Several factors lead to drug shortages, including quality control problems during manufacturing, raw material shortages, regulatory issues, discontinuation of products from the market for safety or economic reasons, procurement barriers, and natural disasters.^17^ The IV fluid shortage caused by Hurricane Helene prompted our health system to enact conservation efforts that successfully reduced IV fluid utilization across multiple service lines. Although there was initial concern among key stakeholders that conservation efforts might adversely impact clinical outcomes, we did not observe significant differences in hypotension, renal replacement therapy, ICU transfer, or mortality. Notably, the intervention focused on decreasing routine IV fluid use (e.g., perioperative maintenance fluid) and did not limit IV fluid resuscitation when appropriate, perhaps leading to more judicious IV fluid utilization without adverse effects.

Next, we observed a significant reduction in loop diuretic utilization during the postintervention period. This finding was not surprising in the context of decreased IV fluid administration. Avoidance of fluid accumulation and fluid overload are important considering their associations with worse clinical outcomes and higher costs.^18^, ^19^, ^20^

The reduction in length of stay by more than half a day is perhaps the most significant and unintended finding of this study. In a pooled analysis of more than 600 hospitals across the United States, Magee et al. reported fluid overload was associated with an increased length of stay.^20^ Although the purpose of our local intervention was to conserve IV fluids, we speculate that the reduced length of stay was a related consequence. It is conceivable that conservative IV fluid administration leads to fewer patients with volume overload, reflected by reduced diuretic utilization, thereby shortening hospitalizations. Results of our sensitivity and subgroup analyses (Supporting Information S1: Appendices E and F) support this hypothesis. We questioned whether the “July effect”^16^ (new graduate medical education trainees) was negatively impacting outcomes during the preintervention period (July to October) relative to the postintervention period (October to January). Using identical methodology, we compared pre‐ and postintervention periods from 1‐year earlier. We did not observe evidence of the “July effect” on IV fluid or diuretic use, nor on length of stay, during the preceding year. We then performed a subgroup analysis demonstrating that the greatest reductions in IV fluid use, diuretic administration, and length of stay occurred in surgical admissions. These observations further support the effectiveness of the IV fluid conservation strategies and related effects, particularly in the perioperative setting.

To contextualize this finding, a 0.6‐day reduction represents an approximately 11% decrease in mean length of stay. At an estimated average cost of $4070 per inpatient day^21^ and an average of 88.7 admissions/day during the preintervention period, this reduction translates to roughly $2442 in savings per hospitalization, or a projected $79.1 million annually at the institutional level. Beyond economic implications, shorter hospitalizations are associated with reduced risk of hospital‐acquired complications, including healthcare‐associated infections,^22^ venous thromboembolism,^23^ and functional decline, particularly among older adults.^24^ These downstream clinical benefits may further amplify the value of interventions that limit unnecessary IV fluid administration.

Although we did observe a small increased risk of acute kidney injury^6^ (OR = 1.1; *p* = .024) following the intervention, it is of uncertain clinical significance. Notably, a significant difference in requiring renal replacement therapy (OR = 0.92; *p* = .625) was not identified postintervention.

Overall, the conservation efforts enacted at our institution appear generally safe and effective in maintaining the quality of care. These findings are particularly informative for other healthcare systems grappling with IV fluid shortages. By highlighting successful strategies for fluid management, our results can serve as a guide to optimize care while ensuring appropriate resource allocation. This study underscores the potential for health systems to implement strategies that may simultaneously improve resource utilization and clinical outcomes when faced with similar adversity. Admittedly, our institutional changes were prompted by a natural disaster, but we challenge health system leaders to examine areas with standardized care delivery (e.g., perioperative IV fluid) to identify opportunities for improvement.

Several study limitations exist. The retrospective, quasi‐experimental (nonrandomized) study design is the greatest weakness. To address this, we used regression modeling and ITS analysis, arguably the strongest quasi‐experimental design for causal inference.^25^ We cannot exclude a Hawthorne effect as the intervention was not blinded. Clinicians may have utilized IV fluids more efficiently without reducing total volumes, although this would not explain the simultaneous reduction in loop diuretic use. Next, all study data were obtained from electronic database queries, and although we aimed to include pertinent patient‐ and admission‐related characteristics to control for confounding, unrecognized differences between study cohorts may exist. Acute kidney injury staging used creatinine‐based kidney disease: improving global outcomes (KDIGO) criteria, as urine output and baseline creatinine were often missing, potentially underestimating incidence. Lastly, the study was performed at a single academic medical center with demographics (e.g., predominantly White, single geographic region in the Mountain West) that may not be representative of other health systems, potentially limiting the generalizability of our findings.

## CONCLUSIONS

We observed significant reductions in IV fluid utilization, diuretic administration, and hospital length of stay following a multifaceted, system‐wide intervention designed to conserve IV fluids during a national shortage. Our findings suggest that health systems implementing similar conservation efforts should carefully consider whether to resume preshortage processes as IV fluid conservation has the potential to improve clinical outcomes.

## CONFLICT OF INTEREST STATEMENT

The authors declare no conflicts of interest.

## Supporting information

Supplementary Information

## Data Availability

The data that support the findings of this study are available on request from the corresponding author. The data are not publicly available due to privacy or ethical restrictions.
