# Supplementary material for: Resource management and clinical outcomes during a national intravenous fluid shortage
Source: J Hosp Med. 2026 May 15;21(11):1244–51. doi: 10.1002/jhm.70352 (PMC13728524; doi:10.1002/jhm.70352)
Supplement: Supplementary file 1 [file JHM-21-1244-s001.docx]

**Appendix A**. Subgroup assignment based on discharging hospital service line.

| **Discharging Service Line** | **Surgical Subgroup** | **Medical Subgroup** | **Ob/Gyn Subgroup** |
| --- | --- | --- | --- |
| General Surgery | X |  |  |
| Orthopedic Surgery | X |  |  |
| Otolaryngology | X |  |  |
| Vascular Surgery | X |  |  |
| Urology | X |  |  |
| Plastic Surgery | X |  |  |
| Cardiothoracic Surgery | X |  |  |
| Neurosurgery | X |  |  |
| Hepatobiliary and Transplant Surgery | X |  |  |
| Internal Medicine |  | X |  |
| Cardiology |  | X |  |
| Hematology |  | X |  |
| Oncology |  | X |  |
| Pulmonary and Critical Care |  | X |  |
| Nephrology |  | X |  |
| Neurology |  | X |  |
| Obstetrics and Gynecology |  |  | X |
| Maternal-Fetal Medicine |  |  | X |
| Gynecology Oncology |  |  | X |

**Appendix B.** Intravenous and Irrigation Fluid Product Usage and Supply Monitoring Dashboard

**Appendix C. ASHP Recommendations for Management of Intravenous Fluid Shortages**

| **Category** | **Recommendation** |
| --- | --- |
| **General Recommendations** | Implement an organization-specific action plan to conserve IV fluids where possible; establish policies to allow interchanges between clinically equivalent products. |
|  | Ensure an interdisciplinary team is making rationing decisions using an ethical framework. |
|  | Establish a schedule to regularly assess restrictions and adjust based on current and anticipated supply. |
|  | Communicate changes in shortage status and action plan adjustments to stakeholders as soon as possible. |
|  | Identify vulnerable patients and populations with specific needs (e.g., pediatric and neonatal patients) and consider policies that reserve or prioritize fluid products for their needs. |
| **Conservation – Large Volume Products** | Evaluate clinical need to continue IV fluid replacement at every shift change and bag change. |
|  | Assess the need to initiate and continue “keep vein open” (KVO) orders at every shift change; consider reducing to the lowest reasonable rate. |
|  | Consider catheter locks with flushes for eligible patients. |
|  | Use oral electrolyte and hydration whenever possible; discontinue infusions as soon as appropriate. |
|  | Avoid discontinuing and restarting IV fluids during transitions of care unless clinically necessary. |
|  | Consider policies allowing completion of a currently hanging infusion bag before switching to a different product unless clinically contraindicated. |
|  | Evaluate total fluid requirements for surgeries and transition to oral fluids within 24 hours post-operatively. |
|  | Develop policies for substitution of IV solutions based on product availability (e.g., Lactated Ringer’s for 0.9% sodium chloride or vice versa). |
|  | Use smaller volume bags for low infusion rates; consider reserving some products for specific clinical situations. |
|  | Avoid opening and “pre-spiking” bags in anticipation of use for surgery. |
|  | Consider extending hang times for IV solutions. Evidence shows no difference in CLABSI rates at 96 hours for fluids administered via central venous catheters. |
|  | Review use of sterile water for rinsing equipment or reconstituting oral solutions; use purified, distilled, or reverse osmosis water per USP Chapter <795> when appropriate. |
| **Conservation – Small Volume Products** | Review use of fluids as supplies (e.g., starting IV lines, blood administration, line flushes); consider single-use flush syringes when possible. |
|  | Switch products to IV push when the patient’s clinical status and drug properties allow, following ISMP Safe Practice Guidelines. |
|  | Adhere to CDC use guidelines for single-use vials. |
|  | Change IV medications and electrolytes to clinically appropriate oral products whenever possible; expand IV-to-oral policies as needed. |
|  | If oral route is not feasible, consider intramuscular or subcutaneous injection; adhere to recommended maximum volumes per injection. |
|  | Consider alternative IV solutions when therapeutic interchange is not clinically important (e.g., maintaining line patency, blood administration). |
|  | Review stock of small volume bags and vials for compatibility with proprietary bag-and-vial systems. |
|  | Do not make practice change plans that will require additional fluids or specific products. |
| **Conservation – Irrigation Solutions** | Review available equivalent and alternative irrigation products. |
|  | Consider using tap water instead of IV or sterile irrigation solutions for wound cleansing when clinically appropriate (per ACEP recommendations). |
|  | Identify sterile fluids being used as cleaning solutions; contact manufacturers to determine if distilled water can be substituted. |
|  | Avoid using sterile water for irrigation for routine reconstitution of oral products or other non-irrigation pharmacy uses. |
|  | Consider whether pre-mixed commercial irrigation solutions can extend irrigation volumes. |
|  | Evaluate whether conserved IV solutions can be repurposed as irrigation solutions; avoid creating secondary shortages of CRRT or inhalation solutions. |
| **Operational Strategies – Large Volume Shortages** | Evaluate supplies on a health system–wide basis to redeploy solutions to areas of greatest need. |
|  | Minimize unit stock of large volume bags; stock only in critical care, procedure, and emergency care areas where fluids are essential. |
|  | Ensure smaller volume bags are stocked in supply areas. |
|  | Work closely with supply chain to obtain accurate system-wide estimates of stock on hand. |
|  | Evaluate whether fluids used to administer secondary infusions are accounted for in overall counts. |
|  | Consider using smaller volume bags or vials for reconstituting drugs in pharmacy instead of liter bags. |
|  | For situations requiring small volumes when small volume packages are unavailable, aliquots drawn into syringes in pharmacy may allow a single bag to serve multiple patients (e.g., via syringe pumps). Follow USP Chapter <797> for beyond-use dating. |
|  | Avoid buying products from sources outside the traditional supply chain. Report suspected illegal activity or price gouging to appropriate authorities. |
| **Operational Strategies – Small Volume Shortages** | Transition to commercially manufactured premix medications when available. |
|  | If switching to IV push, ensure sufficient supplies of diluent vials, syringes, and needles. |
|  | Leverage admixtures available from 503B outsourcers; communicate with outsourcers to understand how their supply may be affected. |
|  | Consider EHR changes to allow use of either dextrose or saline for admixture of drugs compatible with both solutions; use alerts or forced functions when a drug is compatible in only one diluent. |
|  | Implement or encourage barcode scanning of admixture ingredients to ensure correct solution is used and documented. |
|  | Consider preparing and dispensing medications in ready-to-administer syringes for IV push or syringe pump infusion. |
|  | If all other options are exhausted, consider compounding SVPs from commercially available LVPs (e.g., repackaging 1 L bags into 50, 100, or 250 mL bags). Follow USP Chapter <797> for beyond-use dating; thoroughly inspect bags if frozen to extend dating. |
|  | Only compound in containers that adequately reflect the final volume; affix labels to cover printed capacity if container exceeds final volume. |
| **Operational Strategies – Irrigation Shortages** | Identify surgical procedures utilizing the largest irrigation volumes; consider rescheduling when possible. |
|  | Identify all areas of the organization using irrigation fluids; use past purchase data to establish usual rates of use. |
|  | Consider manual counts in OR areas to ensure accurate on-hand quantities. |
|  | Set internal allocations to prevent one area from exceeding its share; consider limiting ordering to a small number of designated personnel. |
|  | Evaluate surgery schedules and usual irrigation volumes needed against quantities on hand to determine if all surgeries can proceed. |
| **Infusion Pumps / Informatics Strategies** | Allocate appropriate clinical informatics resources to manage critical shortages. |
|  | Review order sets and preference lists for default fluid orders, including KVO orders, and update when possible. |
|  | Identify and update individual prescriber preference lists that may have manually overridden order-set defaults. |
|  | Consider alerts to notify clinicians when fluids are ordered for patients tolerating oral hydration. |
|  | Ensure existing pump libraries are up to date; use clinical, safety/quality, and informatics teams to vet any additions or changes through appropriate channels. |
|  | Reflect use of smaller volumes in infusion pump libraries, electronic order sets, and standard fluid labels as needed. |
|  | Review, revise, or develop good infusion pump practices and protocols; consider where ambulatory infusion pumps can be used. |
|  | Maintain standardization whenever possible, especially if the same pumps are used for both adult and pediatric patients. |
| **Communication Strategies** | Communicate practice changes using established channels (e.g., local intranet, EHR alerts, emails, daily huddles, flyers, labeling). |
|  | Ensure clinical informatics team is aware of the need for priority changes in drug records, charge description masters, and infusion pump libraries. |
|  | Consider having physician and nursing champions in addition to the pharmacy lead to assist with communication, practice changes, and stock updates. |
| **Safety Considerations** | Compounding sodium chloride from sterile water and concentrated sodium chloride is labor-intensive and may worsen existing shortages. For urgent sodium and fluid replacement, consider adding concentrated sodium chloride to dextrose or other commercially available LVPs. |
|  | Avoid use of sodium chloride irrigation solution administered intravenously; limits on particulate matter differ between these products. |
|  | Ensure all healthcare professionals administering medications have access to IV push policies and guidelines and have been trained and assessed for competency. Consider an IV push competency assessment tool. |
|  | Do not allow use of “stock” bags that could potentially be used for multiple patients. |
|  | If transitioning medications to syringes for syringe infusion pump administration, ensure staff are adequately trained to use the technology. |

*Abbreviations: ASHP = American Society of Health-System Pharmacists; IV = intravenous; KVO = keep vein open; CLABSI = central line–associated bloodstream infection; USP = United States Pharmacopeia; CDC = Centers for Disease Control and Prevention; ISMP = Institute for Safe Medication Practices; ACEP = American College of Emergency Physicians; EHR = electronic health record; CRRT = continuous renal replacement therapy; SVP = small volume parenteral; LVP = large volume parenteral; OR = operating room.*

*Source: American Society of Health-System Pharmacists. ASHP Guidelines on Managing Drug Product Shortages.*

**Appendix D**. Loop diuretic conversion chart to furosemide equivalent units.

| **Loop Diuretic** | **Oral Administration** | **Parenteral Administration** | **Furosemide Equivalent Units** |
| --- | --- | --- | --- |
| Furosemide | 40mg | 20mg | 40 |
| Torsemide | 20mg | 20mg | 40 |
| Bumetanide | 1mg | 1mg | 40 |

**Appendix E**. Flow diagram of included hospital admissions.

Ob/Gyn Admissions (n=1,143)

Surgical Admissions (n=2,544)

Medical Admissions (n=3,996)

Ob/Gyn

Admissions (n=1,289)

Surgical Admissions (n=2,550)

Medical Admissions (n=4,325)

Post-Intervention Admissions (n=7,683)

Pre-Intervention Admissions (n=8,164)

Total number of inpatient admissions identified

(n=22,358)

Included inpatient admissions

(n=15,847)

Excluded Admissions (n=6,511)

- Newborn Nursery/Neonatology (n=2,692)
- Psychiatry (n=1,991)
- Missing Service Line (n=742)
- Other (n=652)
- Physical Medicine & Rehabilitation (n=425)

**Appendix F.** Sensitivity analysis comparing inpatient admissions and subgroups from similar intervention periods 1-year prior.

| Outcomes | Comparison Cohort - Jul 2023 to Oct 2023 | Comparison Cohort - Oct 2023 to Jan 2024 | *p*-value |
| --- | --- | --- | --- |
|  | (n=7,605) | (n=7,683) |  |
| Inpatient Admissions |  |  |  |
| Bags of IV fluid dispensed during admission, mean (SD) | 5.2 (8.7) | 5.9 (10.9) | <0.001 |
| Length of stay in days, mean (SD) | 5.8 (7.1) | 5.8 (7.5) | 0.937 |
| Any diuretic administered during admission, n (%) | 1,564 (20.6) | 1,508 (19.4) | 0.080 |
| Days of diuretic administration, mean (SD) | 1.0 (3.2) | 1.0 (3.4) | 0.894 |
| Total dose of diuretic received during admission in oral furosemide equivalents, mean (SD) | 128.6 (549.5) | 145.8 (790.6) | 0.119 |
| Average daily dose of diuretic received, mean (SD) | 20.3 (56.7) | 20.0 (57.7) | 0.765 |
| Acute Kidney Injury, n (%) | 733 (10.9) | 807 (11.7) | 0.131 |
| Hypotension during admission, n (%) | 2,327 (30.6) | 2,368 (30.5) | 0.915 |
| ICU Transfers, n (%) | 276 (3.6) | 314 (4.1) | 0.178 |
| 30-Day mortality, n (%) | 346 (4.6) | 361 (4.7) | 0.761 |
| Inhospital morality, n (%) | 198 (2.6) | 200 (2.6) | 0.920 |

| Outcomes | Comparison Cohort - Jul 2023 to Oct 2023 | Comparison Cohort - Oct 2023 to Jan 2024 | *p-*value |
| --- | --- | --- | --- |
|  | (n=2,499) | (n=2,505) |  |
| Surgical Admissions |  |  |  |
| Bags of IV fluid dispensed during admission, mean (SD) | 6.4 (9.0) | 7.6 (13.8) | <0.001 |
| Length of stay in days, mean (SD) | 6.5 (9.0) | 6.4 (9.8) | 0.753 |
| Any diuretic* administered during admission, n (%) | 528 (21.1) | 461 (18.4) | 0.015 |
| Days of diuretic administration, mean (SD) | 1.1 (3.8) | 1.1 (4.0) | 0.536 |
| Total dose of diuretic received during admission in oral furosemide equivalents, mean (SD) | 120.0 (523.7) | 125.0 (851.3) | 0.804 |
| Average daily dose of diuretic received, mean (SD) | 16.2 (41.9) | 14.5 (40.3) | 0.152 |
| Acute Kidney Injury, n (%) | 236 (10.0) | 219 (9.4) | 0.463 |
| Hypotension during admission, n (%) | 723 (28.9) | 739 (29.5) | 0.658 |
| ICU Transfers, n (%) | 82 (3.3) | 82 (3.3) | 0.988 |
| 30-Day mortality, n (%) | 59 (2.4) | 60 (2.4) | 0.937 |
| Inhospital morality, n (%) | 49 (2.0) | 47 (1.9) | 0.827 |

| Outcomes | Comparison Cohort - Jul 2023 to Oct 2023 | Comparison Cohort - Oct 2023 to Jan 2024 | *p*-value |
| --- | --- | --- | --- |
|  | (n=3,951) | (n=4,082) |  |
| Medical Admissions |  |  |  |
| Bags of IV fluid dispensed during admission, mean (SD) | 4.8 (9.4) | 5.5 (10.1) | 0.003 |
| Length of stay in days, mean (SD) | 6.1 (6.3) | 6.2 (6.4) | 0.426 |
| Any diuretic* administered during admission, n (%) | 1,004 (25.4) | 1,018 (25.0) | 0.626 |
| Days of diuretic administration, mean (SD) | 1.2 (3.2) | 1.3 (3.4) | 0.469 |
| Total dose of diuretic received during admission in oral furosemide equivalents, mean (SD) | 170.9 (633.3) | 199.3 (856.8) | 0.091 |
| Average daily dose of diuretic received, mean (SD) | 28.6 (69.8) | 28.9 (71.5) | 0.838 |
| Acute Kidney Injury, n (%) | 487 (12.6) | 579 (14.4) | 0.018 |
| Hypotension during admission, n (%) | 1,245 (31.5) | 1,272 (31.2) | 0.735 |
| ICU Transfers, n (%) | 190 (4.8) | 222 (5.4) | 0.201 |
| 30-Day mortality, n (%) | 286 (7.2) | 301 (7.4) | 0.816 |
| Inhospital morality, n (%) | 149 (3.8) | 153 (3.8) | 0.957 |

| Outcomes | Comparison Cohort - Jul 2023 to Oct 2023 | Comparison Cohort - Oct 2023 to Jan 2024 | *p-*value |
| --- | --- | --- | --- |
|  | (n=1,155) | (n=1,172) |  |
| Ob/Gyn Admissions |  |  |  |
| Bags of IV fluid dispensed during admission, mean (SD) | 4.0 (3.1) | 3.8 (3.2) | 0.107 |
| Length of stay in days, mean (SD) | 3.2 (3.1) | 3.0 (3.6) | 0.276 |
| Any diuretic* administered during admission, n (%) | 32 (2.8) | 29 (2.5) | 0.655 |
| Days of diuretic administration, mean (SD) | 0.07 (0.68) | 0.07 (0.52) | 0.939 |
| Total dose of diuretic received during admission in oral furosemide equivalents, mean (SD) | 2.8 (28.4) | 3.7 (39.7) | 0.523 |
| Average daily dose of diuretic received, mean (SD) | 1.1 (7.4) | 1.1 (8.6) | 0.485 |
| Acute Kidney Injury, n (%) | 10 (2.0) | 9 (1.7) | 0.707 |
| Hypotension during admission, n (%) | 359 (31.1) | 357 (30.5) | 0.745 |
| ICU Transfers, n (%) | 4 (0.4) | 10 (0.9) | 0.114 |
| 30-Day mortality, n (%) | 1 (0.1) | 0 (0) | 0.314 |
| Inhospital morality, n (%) | 0 (0) | 0 (0) | n/a |

**Appendix G.** Baseline characteristics and adjusted clinical outcomes by study subgroups.

| Subgroup Characteristic | Pre-Intervention Period | Post-Intervention Period | p-value |
| --- | --- | --- | --- |
|  | (N= 8164) | (N=7683) |  |
|  |  |  |  |
| **Surgical Admissions** | (n=2550) | (n=2544) |  |
| Age in years, mean (SD) | 55.2 (18.6) | 56.1 (18.0) | 0.074 |
| Female, n (%) | 1,128 (44.2) | 1,182 (46.5) | 0.11 |
| Race/Ethnicity, n (%) |  |  | 0.317 |
| *White/Caucasian* | 2067 (81.1) | 2034 (80.1) |  |
| *Latin-x/Hispanic* | 229 (9.0) | 240 (9.5) |  |
| *Black/African American* | 33 (1.3) | 27 (1.1) |  |
| *Unidentified* | 84 (3.3) | 101 (4.0) |  |
| BMI in kg/m2, mean (SD) | 28.7 (7.4) | 29.1 (7.3) | 0.049 |
| Charlson Comorbidlity Index, mean (SD) | 2.8 (2.9) | 2.8 (2.8) | 0.54 |
| MS-DRG weight, mean (SD) | 3.2 (3.4) | 3.1 (3.1) | 0.064 |
|  |  |  |  |
| **Medical Admissions** | (n=4325) | (n=3996) |  |
| Age in years, mean (SD) | 57.4 (18.5) | 57.7 (18.3) | 0.485 |
| Female, n (%) | 2,061 (47.7) | 1,828 (45.8) | 0.081 |
| Race/Ethnicity, n (%) |  |  | <0.001 |
| *White/Caucasian* | 3236 (74.9) | 3003 (75.2) |  |
| *Latin-x/Hispanic* | 446 (10.3) | 367 (9.2) |  |
| *Black/African American* | 120 (2.8) | 114 (2.9) |  |
| *Unidentified* | 163 (3.8) | 214 (5.4) |  |
| BMI in kg/m2, mean (SD) | 28.4 (8.1) | 28.5 (7.8) | 0.767 |
| Charlson Comorbidlity Index, mean (SD) | 4.3 (3.4) | 4.2 (3.4) | 0.286 |
| MS-DRG weight, mean (SD) | 2.0 (2.8) | 2.0 (1.9) | 0.811 |
|  |  |  |  |
| **Ob/Gyn Admissions** | (n=1289) | (n=1143) |  |
| Age in years, mean (SD) | 31.3 (8.6) | 31.5 (8.8) | 0.578 |
| Female, n (%) | 1,285 (99.7) | 1,137 (99.5) | 0.409 |
| Race/Ethnicity, n (%) |  |  | 0.316 |
| *White/Caucasian* | 746 (58.9) | 665 (59.1) |  |
| *Latin-x/Hispanic* | 212 (16.8) | 206 (18.3) |  |
| *Black/African American* | 59 (4.7) | 34 (3.0) |  |
| *Unidentified* | 78 (6.2) | 80 (7.1) |  |
| BMI in kg/m2, mean (SD) | 32.3 (7.6) | 32.4 (7.6) | 0.614 |
| Charlson Comorbidlity Index, mean (SD) | 0.48 (1.3) | 0.51 (1.3) | 0.602 |
| MS-DRG weight, mean (SD) | 0.9 (0.4) | 0.9 (0.4) | 0.918 |

Abbreviations: SD = standard deviation; BMI = body mass index; MS-DRG= Medicare Severity Diagnosis Related Group; Ob/Gyn= Obstetrics and Gynecology

| **Outcomes** | **Pre-Intervention** | **Post-Intervention** | **p*-*value** |  |
| --- | --- | --- | --- | --- |
|  |  |  |  |  |
| **Surgical Admissions** | (n=2550) | (n=2544) |  |  |
| Resource Utilization |  |  |  |  |
| Bags of IV fluid dispensed, mean (95% CI) | 6.9 (6.6 - 7.2) | 4.2 (4.0 - 4.4) | <0.001 |  |
| Clinical Outcomes |  |  |  |  |
| Length of stay in days, mean (95% CI) | 5.4 (5.2 - 5.6) | 4.8 (4.6 - 4.9) | <0.001 |  |
| Loop diuretic administered during admission, OR (95% CI) | ref | 0.78 (0.66 - 0.92) | 0.003 |  |
| Days of diuretic administration, mean (95% CI) | 0.51 (0.44 - 0.59) | 0.40 (0.33 - 0.46) | 0.017 |  |
| Total dose of diuretic administered in oral furosemide equivalents, mean (95% CI) | 44.6 (34.4 - 54.7) | 32.2 (23.6 - 40.9) | 0.047 |  |
| Average daily dose of diuretic, mean (95% CI) | 10.6 (9.2 - 12.0) | 7.7 (6.4 - 9.0) | 0.004 |  |
| Acute kidney injury, OR (95% CI) | ref | 1.17 (0.95 - 1.43) | 0.14 |  |
| Severe acute kidney injury, OR (95% CI) | ref | 0.97 (0.69 – 1.37) | 0.867 |  |
| Renal Replacement Therapy, OR (95% CI) | ref | 0.99 (0.44 - 2.19) | 0.973 |  |
| Hypotension, OR (95% CI) | ref | 1.0 (0.9 -1.2) | 0.654 |  |
| ICU Transfer, OR (95% CI) | ref | 0.85 (0.61 - 1.18) | 0.328 |  |
| Post-operative atrial fibrillation*, OR (95% CI) | ref | 0.87 (0.65 - 1.16) | 0.347 |  |
| 30-day mortality, OR (95% CI) | ref | 0.65 (0.41 - 1.03) | 0.067 |  |
| Inhospital morality, OR (95% CI) | ref | 0.57 (0.32 - 1.02) | 0.059 |  |
|  |  |  |  |  |
| **Medical Admissions** | (n=4325) | (n=3996) |  |  |
| Resource Utilization |  |  |  |  |
| Bags of IV fluid dispensed, mean (95% CI) | 5.0 (4.8 - 5.3) | 3.3 (3.2 - 3.5) | <0.001 |  |
| Clinical Outcomes |  |  |  |  |
| Length of stay in days, mean (95% CI) | 6.1 (5.9 - 6.3) | 5.6 (5.4 - 5.7) | <0.001 |  |
| Loop diuretic administered during admission, OR (95% CI) | ref | 0.94 (0.84 - 1.04) | 0.232 |  |
| Days of diuretic administration, mean (95% CI) | 0.82 (0.74 - 0.90) | 0.78 (0.70 - 0.86) | 0.476 |  |
| Total dose of diuretic administered in oral furosemide equivalents, mean (95% CI) | 96.2 (83.6 - 108.7) | 94.5 (78.6 - 110.3) | 0.869 |  |
| Average daily dose of diuretic, mean (95% CI) | 18.3 (16.7 - 20.0) | 18.3 (16.4 - 20.1) | 0.944 |  |
| Acute kidney injury, OR (95% CI) | ref | 1.0 (0.99 - 1.01) | 0.342 |  |
| Severe acute kidney injury, OR (95% CI) | ref | 0.92 (0.75 – 1.12) | 0.413 |  |
| Renal Replacement Therapy, OR (95% CI) | ref | 0.92 (0.66 - 1.28) | 0.625 |  |
| Hypotension, OR (95% CI) | ref | 1.0 (0.99 - 1.01) | 0.557 |  |
| ICU Transfer, OR (95% CI) | ref | 1.0 (0.99 - 1.01) | 0.126 |  |
| Post-operative atrial fibrillation*, OR (95% CI) | ref | 1.02 (1.01 - 1.03) | 0.757 |  |
| 30-day mortality, OR (95% CI) | ref | 1.02 (1.01 - 1.03) | 0.448 |  |

| **Ob/Gyn Admissions** | (n=1289) | (n=1143) |  |  |
| --- | --- | --- | --- | --- |
| Resource Utilization |  |  |  |  |
| Bags of IV fluid dispensed, mean (95% CI) | 3.9 (3.8 - 4.1) | 2.4 (2.3 - 2.6) | <0.001 |  |
| Clinical Outcomes |  |  |  |  |
| Length of stay in days, mean (95% CI) | 3.1 (2.9 - 3.2) | 3.0 (2.9 - 3.2) | 0.735 |  |
| Loop diuretic administered during admission, OR (95% CI) | ref | 0.57 (0.27 - 1.19) | 0.133 |  |
| Days of diuretic administration, mean (95% CI) | 0.03 (-0.01 - 0.07) | 0.002 (-0.001 - 0.007) | 0.032 |  |
| Total dose of diuretic administered in oral furosemide equivalents, mean (95% CI) | 0.99 (-0.13 - 2.10) | 0.16 (-0.06 - 0.38) | 0.065 |  |
| Average daily dose of diuretic, mean (95% CI) | 0.33 (-0.02 - 0.67) | 0.09 (-0.04 - 0.22) | 0.2 |  |
| Acute kidney injury, OR (95% CI) | ref | 2.1 (0.9 - 5.1) | 0.108 |  |
| Severe acute kidney injury, OR (95% CI) | ref | - | - |  |
| Renal Replacement Therapy, OR (95% CI) | ref | - | - |  |
| Hypotension, OR (95% CI) | ref | 1.1 (0.9 - 1.3) | 0.339 |  |
| ICU Transfer, OR (95% CI) | ref | 0.48 (0.09 - 2.52) | 0.382 |  |
| Post-operative atrial fibrillation*, OR (95% CI) | ref | - | - |  |
| 30-day mortality, OR (95% CI) | ref | - | - |  |

Abbreviations: IV = intravenous; CI = confidence interval; OR = odds ratio; ICU = intensive care unit

**Supplementary Figure 1. Interrupted time series analysis of weekly IV fluid use for Medical Admissions subgroup.**

ß_1_ = 0.04 [0.002, 0.08]; p=0.039

ß_2_ = -0.11 [-0.74, 0.51]; p=0.710

ß_3_ = -0.10 [-0.21, 0.01]; p=0.074

ß_1_= pre-intervention weekly trend; ß_2_= level change at time of intervention; ß_3_= post-intervention weekly trend.

**Supplementary Figure 2. Interrupted time series analysis of weekly IV fluid use for Ob/Gyn Admissions subgroup.**

ß_1_ = 0.01 [-0.02, 0.04]; p=0.570

ß_2_ = -0.59 [-1.31, 0.14]; p=0.106

ß_3_ = -0.09 [-0.18, 0.01]; p=0.072

ß_1_= pre-intervention weekly trend; ß_2_= level change at time of intervention; ß_3_= post-intervention weekly trend.

**Supplementary Figure 3. Interrupted time series analysis of weekly IV fluid use for Surgical Admissions subgroup.**

ß_1_ = 0.07 [0.02, 0.11]; p=0.003

ß_2_ = -0.97 [-1.64, -0.31]; p=0.006

ß_3_ = -0.12 [-0.20, -0.03]; p=0.009

ß_1_= pre-intervention weekly trend; ß_2_= level change at time of intervention; ß_3_= post-intervention weekly trend.
